# Repetitive transcranial magnetic stimulation focusing on patients with neuropathic pain in the upper limb: a randomized sham-controlled parallel trial

**DOI:** 10.1038/s41598-024-62018-x

**Published:** 2024-05-23

**Authors:** Nobuhiko Mori, Koichi Hosomi, Asaya Nishi, Akimitsu Miyake, Tomomi Yamada, Akiyoshi Matsugi, Yasutomo Jono, Chanseok Lim, Hui Ming Khoo, Naoki Tani, Satoru Oshino, Youichi Saitoh, Haruhiko Kishima

**Affiliations:** 1 Department of Neurosurgery, Osaka University Graduate School of Medicine, 2-2 Yamadaoka, Suita, Osaka 565-0871 Japan; 2 Department of Neurosurgery, Toyonaka Municipal Hospital, Toyonaka, Japan; 3 Department of Medical Innovation, Osaka University Hospital, Suita, Japan; 4 Department of AI and Innovative Medicine, Tohoku University Graduate School of Medicine, Sendai, Japan; 5 Faculty of Rehabilitation, Shijonawate Gakuen University, Daitou, Japan; 6 Faculty of Health Sciences, Naragakuen University, Nara, Japan; 7 Center for Information and Neural Networks (CiNet), Advanced ICT Research Institute, National Institute of Information and Communications Technology (NICT), Suita, Japan; 8 Department of Mechanical Science and Bioengineering, Osaka University Graduate School of Engineering Science, Toyonaka, Japan; 9 Tokuyukai Rehabilitation Clinic, Toyonaka, Japan

**Keywords:** Chronic pain, Pain, Neuropathic pain

## Abstract

This study aimed to evaluate the efficacy and safety of navigation-guided repetitive transcranial magnetic stimulation (rTMS) over the primary motor cortex in patients with neuropathic pain in the upper limb. This randomized, blinded, sham-controlled, parallel trial included a rTMS protocol (10-Hz, 2000 pulses/session) consisting of five daily sessions, followed by one session per week for the next seven weeks. Pain intensity, as well as pain-related disability, quality of life, and psychological status, were assessed. For the primary outcome, pain intensity was measured daily using a numerical rating scale as a pain diary. Thirty patients were randomly assigned to the active rTMS or sham-stimulation groups. In the primary outcome, the decrease (least square [LS] mean ± standard error) in the weekly average of a pain diary at week 9 compared to the baseline was 0.84 ± 0.31 in the active rTMS group and 0.58 ± 0.29 in the sham group (LS mean difference, 0.26; 95% confidence interval, − 0.60 to 1.13). There was no significant effect on the interaction between the treatment group and time point. Pain-related disability score improved, but other assessments showed no differences. No serious adverse events were observed. This study did not show significant pain relief; however, active rTMS tended to provide better results than sham. rTMS has the potential to improve pain-related disability in addition to pain relief.

Clinical Trial Registration number: jRCTs052190110 (20/02/2020).

## Introduction

Neuropathic pain is defined as "pain arising as a direct consequence of a lesion or disease affecting the somatosensory system"^1,2^ and it is estimated to affect approximately 10% of the general population^3,4^. Although several drugs are available for neuropathic pain, their efficacy is limited their side effects may prevent the continuation of medication^5^, and there are an increasing number of cases of addiction and overdose resulting from the misuse or inappropriate prescription of opioids for chronic pain which has become a serious crisis in some countries^6,7^. While surgical treatments, such as electrical motor cortex stimulation, spinal cord stimulation, and deep brain stimulation, are also offered for neuropathic pain^8–11^, these surgical treatments may be ineffective in some cases and may cause complications because of their invasiveness. Over the last several decades, noninvasive brain stimulation therapies, such as repetitive transcranial magnetic stimulation (rTMS), have been proposed as treatment options instead of pharmacological and invasive treatments for chronic pain^12–14^.

In recent systematic reviews and guidelines, high-frequency rTMS over the primary motor cortex (M1) for neuropathic pain has been shown to have a small short-term reduction in pain intensity^15–17^. We have also reported the results of rTMS over M1 for neuropathic pain in some clinical trials^18–25^. Subsequently, a multicenter, randomized, blinded, sham-controlled, parallel trial was conducted to obtain Japanese regulatory approval for the clinical use of medical devices^23^. The results were negative, with no significant difference in both primary and secondary outcomes between the two groups, which were considered to be affected by an insufficient period and dose of intervention, as well as inappropriate patient indications. Subgroup analysis suggested that the analgesic effect of rTMS in patients with upper limb pain was superior to that in patients with facial or lower limb pain. Similarly, another study reported that the analgesic effect of rTMS for upper limb pain tended to be higher than that for lower limb pain^20^. Furthermore, based on the results of a merged analysis of three previous randomized controlled trials (RCTs) conducted at our center, the efficacy of rTMS for neuropathic pain was confirmed to be better for upper limb pain than for facial and lower limb pain^26^. Some previous rTMS studies limited the inclusion criteria to patients with predominantly upper limb pain because the M1 hand area is more easily identifiable as a target for stimulation^27–29^. Moreover, we tested the pain-relieving effect of different doses of stimulation and found that 10-Hz rTMS with 3000 pulses per session was better than 5-Hz with 500 pulses^25^. Therefore, we hypothesized that a higher dose per session of rTMS for patients with neuropathic pain in the upper limbs would be more appropriate for the rTMS protocol and target population.

The short-term analgesic efficacy of rTMS has already been described; however, it is not clinically beneficial and is one of the challenges to overcome for pain treatment with rTMS. With respect to this challenge, clinical studies have been conducted with the promise of a cumulative effect of long-term administration of rTMS on pain relief. Pain relief after the long-term administration of rTMS (4–15 sessions for approximately 12 months) for central neuropathic pain has been reported in some open-label studies^30–32^. In addition, the aforementioned RCT reported that patients enrolled in the additional 4 weekly sessions after one-week daily session achieved more pain relief^23^. Recently, rTMS over M1 for 22 weeks for peripheral neuropathic pain revealed better pain relief than the sham^33^. These findings suggest that long-term rTMS may provide more clinically meaningful pain relief than single-session or short-term interventions. Therefore, this study was positioned as a pilot trial for future large-scale trials and evaluated the efficacy and safety of an eight-week administration of 10-Hz rTMS to the M1 hand area, focusing on the target population with neuropathic pain in the upper limbs.

## Materials and methods

### Study design and trial overview

A randomized, patient- and assessor-blinded, sham-controlled, parallel trial at the Osaka University Hospital was performed. All data management, monitoring, and statistical analyses were performed by an independent academic research organization (the Department of Medical Innovation, Osaka University Hospital). The trial protocol and statistical analysis plan were established prior to the start of the trial and statistical analysis, respectively. The data were captured using an electronic data capture (EDC) system (REDCap; Vanderbilt University, USA). This study was approved by the Ethics Committee of Osaka University Hospital (approval number: S19010) and written informed consent was obtained from all patients participating in the study. This trial was registered with the Japan Registry of Clinical Trials (number jRCTs052190110; 20/02/2020; https://jrct.niph.go.jp/en-latest-detail/jRCTs052190110) and conducted under a rigorous framework in accordance with the Clinical Research Act in Japan.

**Figure 1:**
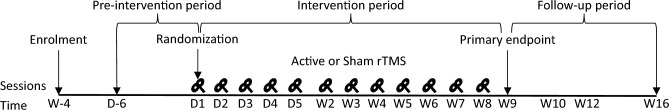
Time schedule of the study. A pain diary was kept every morning at home to evaluate the average pain intensity over the past 24 h from the pre-intervention period to one week after the end of the intervention (week 9). D, day; rTMS, repetitive transcranial magnetic stimulation; W, week.

All patients who provided consent were assessed for eligibility for at least 28 days prior to randomization. Eligible patients were randomly assigned to either the active rTMS or sham stimulation group. The rTMS protocol consisted of daily sessions administered over five consecutive days (induction phase), followed by a maintenance phase that consisted of one session per week for seven weeks, leading to a total of 12 sessions over 8 weeks. An 8-week follow-up period was established after the intervention (Fig. 1; see details in Supplemental Fig. S1). The primary outcome measure was a decrease in pain intensity.

### Patients

Patients with upper limb neuropathic pain diagnosed by a neurosurgeon (KH) specializing in neuropathic pain were recruited from among outpatients of the Department of Neurosurgery at Osaka University Hospital. Patient inclusion criteria were as follows; (1) probable or definite neuropathic pain diagnosed based on the Neuropathic Pain Grading System^1,2,34^, (2) upper limb pain for 6 months or longer, (3) 40–94 mm score using a visual analog scale (VAS) of pain intensity (scaled 0–100 mm), (4) a weekly average of a pain diary (scaled 0–10) of ≥ 4, (5) insufficient pain relief despite receiving drugs for neuropathic pain, and (6) age 20 years or older. Exclusion criteria were inability to complete the written questionnaires, dementia, severe aphasia, major psychiatric disorder, suicidal ideation, pregnancy, history of epilepsy, complete paralysis of the stimulus target upper limbs, or contraindication to TMS (e.g., implanted cardiac pacemaker)^35–37^. The patients’ current pain medication regimens (Supplementary Table S1) were confirmed, and they were asked not to change their medications for neuropathic pain during the study period.

### Randomization and blinding

The patients were randomly assigned in a 1:1 ratio using the assignment function of the EDC system. Stratified permuted block randomization was applied for the assignment, with a weekly average of the pain diary before the intervention (< 6 or ≥ 6) as the assignment factor. The knowledge of the treatment group assignments was limited to the research staff administering the intervention. Treatment group information was stored in a lockable safe and maintained with a password for the EDC system. The patients and assessors were blinded and the assignments were not disclosed during the trial. To ensure blinding, none of the research staff switched roles from blinded to non-blinded, or vice versa. The sham stimulation procedure mimicked that of active rTMS stimulation as much as possible (see the section below for details on the interventions).

### Intervention

Active rTMS was performed using a stimulator (MagPro X100; MagVenture, Denmark) that induced biphasic magnetic pulses using a figure-of-eight coil (MC-B70; MagVenture). Sham stimulation was delivered using a sham coil (MC-P-B70; MagVenture) that mimicked the active coil visually and audibly but delivered no significant magnetic stimulation. During all the sessions, the patients were seated in a comfortable reclining chair. The center of the coil was placed over the M1 hand area, contralateral to the painful side. The coil was positioned tangential to the scalp and perpendicular to the central sulcus, with the handle oriented posterolaterally. The current induced in the brain is oriented in the posterior-anterior direction in the first and third phases of the stimuli and the anterior–posterior direction in the second phase (main phase) of the stimuli^18,24,25,38^. A TMS navigation system (Brainsight; Rogue Research, Inc., Montreal, QC, Canada) was used to accurately monitor the positioning and direction of the coil throughout each session and across sessions^18,24,25^.

Prior to the first session, the target stimulation site was determined by identifying the motor hotspot that induced the most prominent muscle twitch in the hands. The resting motor threshold (RMT), defined as the minimum intensity required to induce one visible muscle twitch, was measured and corresponded to the RMT measured using motor-evoked potentials (MEPs)^20,39^. MEP was not measured, and muscle twitches were adopted instead of MEP because it is more practical in clinical practice and a common method of rTMS therapy for depression. The active rTMS session consisted of 40 trains of TMS pulses delivered at 10 Hz for 5 s (50 pulses/train) with a 25-s inter-train interval, resulting in 2000 pulses per session for a total duration of 20 min. The stimulation intensity was set at 90% of the RMT. If muscle twitches could not be elicited or the RMT was higher than 86% of the maximum stimulator output, the treatment stimulus intensity was set to 77% of the maximum stimulator output, according to the maximum stimulation intensity of previous trials^18–20,23^. The protocol for active rTMS in the current study was developed in accordance with the guidelines for the safe use of rTMS^36,37^. For the sham condition, surface cup electrodes were placed over the M1-hand contralateral to the painful side, and electrical stimulation of 5-mA intensity with a 1-ms pulse duration driven by an electrical stimulator (NS-101, UNIQUE MEDICAL, Tokyo, Japan) was simultaneously delivered with magnetic discharges through the sham coil^24,40^. The pulse frequency, train duration, inter-train interval, and number of trains in the sham group were the same as those in the active rTMS group.

### Assessments and outcomes

Pain intensity was assessed using a numerical rating scale (NRS) as a pain diary (scaled 0–10, 0 = no pain, 10 = maximal pain), VAS (scaled 0–100, 0 = no pain, 100 = maximal pain), and the Japanese version of the short-form McGill pain questionnaire 2 (SF-MPQ2; scaled 0–220, with 4 subscales: continuous pain, intermittent pain, neuropathic pain, and affective descriptors)^41^. For the baseline assessment, patients were asked to evaluate the average pain intensity over the previous 24 h at home every morning for one week before the intervention (pre-intervention period) using a pain diary, VAS, and SF-MPQ. To accurately evaluate the efficacy of the intervention, patients were asked to record the average pain intensity over the past 24 h in a pain diary at home until one week after the end of the intervention (week 9). VAS and SF-MPQ2 scores were also assessed for average pain over the past 24 h immediately before each intervention during the intervention period at weeks 9, 10, 12, and 16. Immediately after each intervention, the VAS score was used to assess the current pain.

The Patient Global Impression of Change (PGIC)^42^, which is a 7-point scale ranging from “very much improved” to “very much worse,” was assessed after the intervention at day 5 and week 9 or the time of withdrawal. The Mini-Mental State Examination (MMSE; scaled 0–30, with higher scores indicating normal cognition) and the Beck Depression Inventory second version (BDI-II; scaled 0–63, with higher scores indicating greater depression)^43^ were evaluated at the screening time point and week 9. The European Quality of Life-5 Dimensions 5-level (EQ-5D-5L; an instrument including an index value scaled 0–1, with higher scores indicating higher quality of life (QOL))^44^, the Pain Catastrophizing Scale (PCS; scaled 0–52, with higher scores indicating higher catastrophizing)^45,46^, and the Pain Disability Assessment Scale (PDAS; scaled 0–60, with higher scores indicating lower pain-related disability)^47^ were used before the intervention on days 1 and 9. At week 9. Blinding was evaluated using the following options: thought active rTMS stimulation, thought sham stimulation, or didn’t know either. All the assessment items were written in a unified form. In the safety assessment, adverse events were defined as signs of undesirable or unintended disease conditions or disorders that occurred in the patient during the device usage. The occurrence of adverse events was collected and evaluated by blinded assessors over the entire study period as well as any cause of intervention discontinuation.

The primary outcome was a decrease in the weekly average of the pain diary at each time point compared to the baseline pain diary. The baseline was calculated as the average pain diary from 6 days before the intervention to the morning of the first day of the intervention (a total of 7 days). Secondary outcomes were VAS_pre_, SF-MPQ2, PGIC, PCS, PDAS, and BDI-II decrease, as well as EQ-5D-5L and MMSE increase. The VAS_pre_ and SF-MPQ2 decreases were calculated as the values of the VAS and SF-MPQ2 immediately before the intervention at each time point compared to the baseline weekly average. The baselines for the VAS and SF-MPQ2 were calculated in the same manner as for the pain diary described above. PCS, PDAS, and BDI-II decrease, as well as EQ-5D-5L and MMSE increase, were calculated as the decrease or increase in values at week 9 compared with the baseline values. The number and percentage of patients who showed improvement in pain diary, VAS_pre,_ and SF-MPQ2 scores at week 9 were also obtained. Thresholds of “improvement” were determined based on the clinical importance of changes: 2 or 20- and 4 or 40-mm decreases correspond to minimally and moderately important decreases in pain diary or VAS_pre_, respectively^42^. Considering the non-invasive nature of rTMS, we set these lower thresholds. Additionally, the VAS score immediately after the intervention in the induction phase (VAS_post_) was used to predict efficacy at week 9 in the active rTMS group.

### Statistical analyses

Based on the results of our previous randomized controlled trial^23^, the average reduction in VAS pain intensity was 38.8 for active rTMS. The effect of sham stimulation was assumed to be half that of active rTMS and the between-group difference was estimated to be 19.4. From the values of these two groups, standard deviations were calculated to be 12.5 for active rTMS and 17.6 for sham group. Therefore, a total of 30 patients (15 patients per group) were estimated to achieve 80% power at a two-sided significance level of 0.05, based on a 7% dropout rate.

The analysis in this study was based on the intention-to-treat principle. Missing data are handled without imputation. Safety analyses were performed for all patients who underwent at least one rTMS session (safety population). For the main analyses of the pain diary, VAS_pre_ and SF-MPQ2, mixed-effects models were employed for repeated measurement (MMRM) with the decrease in each pain score at each time point (weeks 2–9) as a response variable; treatment group, time point, and interaction between treatment group and time point; the baseline weekly average as fixed effects; and patients as a random effect. In each group, a paired t-test without multiple comparison corrections was used to compare the differences between the prior (baseline) and each time point. A correlation analysis was conducted to investigate the relationship between the response to treatment during the induction phase and efficacy at week 9 in the active rTMS group. For response to treatment during the induction phase, the decrease in the average VAS score immediately after intervention (day 1, day 1–2, day 1–3, day 1–4, and day 1–5) relative to baseline were used as VAS_post_. A 2-sample t-test was used to compare the changes in continuous variables between the active rTMS and sham stimulation groups. To compare the two groups, the Wilcoxon rank–sum test was used for ordered categorical variables and Fisher’s exact test for binary variables. As this was an exploratory study, multiple comparison corrections were not performed. To assess the influence of background factors, an analysis of variance (ANOVA) was conducted in subgroup analyses with the decrease in the weekly average of the pain diary (NRS) after 8 weeks of the intervention relative to baseline as the response variable, and the treatment group, time point, and interaction between the treatment group and time point as a fixed effect. In general, an NRS score of 6 and a VAS score of 60 mm correspond to the cutoff point between moderate and severe pain^48^. Between-group comparisons of changes in pain-related disability, cognitive function, QOL, and psychological status (i.e., PCS, PDAS, and BDI-II decrease, as well as EQ-5D-5L and MMSE increase as response variables; treatment group as fixed effects; and preintervention values as covariates) were conducted using an analysis of covariance.

## Results

**Figure 2:**
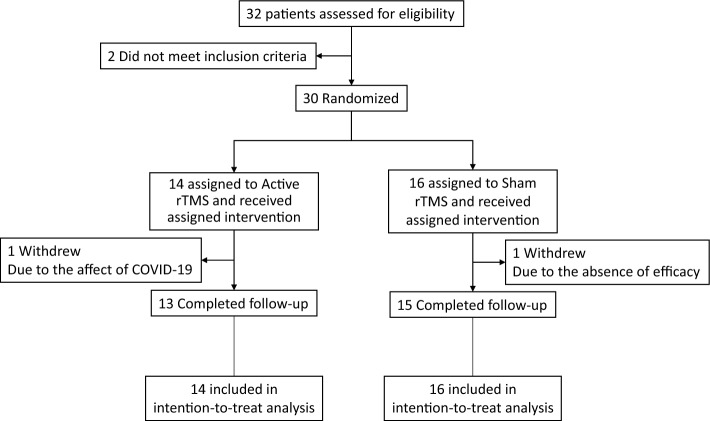
Flow diagram.

Between March 2, 2020, and August 3, 2021, 32 patients were enrolled. Two patients did not meet the inclusion criteria and were excluded, after which the 30 patients were randomly assigned to two groups and received their allocated intervention. Fourteen patients were assigned to the active rTMS group and 16 to the sham stimulation group; two of them (one in the active rTMS group and one in the sham stimulation group) withdrew after the end of the second week of intervention (one due to the COVID-19 epidemic and the other due to the absence of efficacy) (Fig. 2). Table 1 presents the patients’ baseline demographic and clinical characteristics for each group in the intention-to-treat population. No significant differences were observed between the two groups in terms of demographic variables or clinical characteristics.

**Table 1:** Patients’ characteristics at baseline (N = 30).

	Total (30)	Active (n = 14)	Sham (n = 16)
Age (y)	67.0 (9.7)	66.6 (9.2)	67.4 (10.4)
Male sex	19 (63%)	9 (64%)	10 (63%)
Pain duration (month), median (interquartile range)	68 (36–126)	70 (40–139)	65 (23–123)
Cause of pain			
Central neuropathic pain	25 (83%)	11 (79%)	14 (87.5%)
Central post-stroke pain	15	7	8
Spinal cord injury	5	2	3
Other spinal lesion	5	2	3
Peripheral neuropathic pain	5 (17%)	3 (21%)	2 (12.5%)
Postherpetic neuralgia	1	1	0
Phantom limb pain	1	1	0
Root avulsion	1	1	0
Complex regional pain syndrome	1	0	1
Peripheral nerve injury	1	0	1
Location of pain			
Bilateral, left, right	12, 8, 10	4, 5, 5	8, 3, 5
Face	12	4	8
Upper limb	30	14	16
Trunk	7	1	6
Lowe limb	13	4	9
Treated painful region			
Right upper limb	15	7	8
Motor disturbance* (normal-mild, moderate-severe)	22, 8	10, 4	12, 4
Sensory disturbance (normal-mild, moderate-severe)	7, 23	4, 10	3, 13
Allodynia	19 (63%)	9 (64%)	10 (63%)
Weekly average of pain diary (0–10)	6.8 (1.2)	6.7 (1.0)	6.9 (1.3)
VAS of pain intensity (0-100 mm)	68.3 (13.0)	67.6 (12.1)	68.9 (14.0)
SF-MPQ-2 (0–220)	80.5 (48.2)	72.4 (50.9)	87.5 (46.2)
Continuous pain (0–10)	4.0 (2.2)	3.5 (2.5)	4.3 (1.9)
Intermittent pain (0–10)	3.5 (2.5)	3.3 (2.7)	3.7 (2.4)
Neuropathic pain (0–10)	4.1 (2.1)	3.8 (2.2)	4.4 (2.0)
Affective descriptors (0–10)	2.7 (2.6)	2.2 (2.7)	3.2 (2.6)
PDAS (0–60)	23.3 (13.9)	24.8 (14.2)	22 (13.9)
EQ-5D-5L (0–1)	0.551 (0.185)	0.586 (0.160)	0.521 (0.2040)
PCS (0–52)	30.7 (9.1)	30.6 (9.4)	30.8 (9.2)
Rumination (0–20)	15.1 (3.7)	14.6 (3.8)	15.6 (3.8)
Helplessness (0–20)	10.5 (4.0)	10.5 (4.7)	10.6 (3.4)
Magnification (0–12)	5.1 (2.9)	5.5 (3.1)	4.7 (2.8)
BDI-II (0–63)	14.7 (8.8)	12.9 (9.9)	16.4 (7.7)
MMSE (0–30)	29.2 (1.5)	28.9 (1.7)	29.4 (1.3)

Data are presented as mean (standard deviation) or number (%), unless otherwise indicated. Numbers in parentheses after the scoring systems indicate the range of possible scores. The 4 subscales of the SF-MPQ2 are shown as mean scores.

NRS, Numerical rating scale; VAS, visual analog scale; SF-MPQ2, Short-Form McGill Pain Questionnaire 2; PDAS, Pain Disability Assessment Scale; EQ-5D, EuroQol-5 Dimension; PCS, Pain Catastrophizing Scale; BDI-II, Beck Depression Inventory Version 2 ; MMSE, Mini-Mental State Examination.

*Normal to mild motor deficit was defined as muscle strength of grade 4 or more on the painful side, in accordance with Medical Research Council scores.

### Pain dairy (primary outcome)

**Figure 3:**
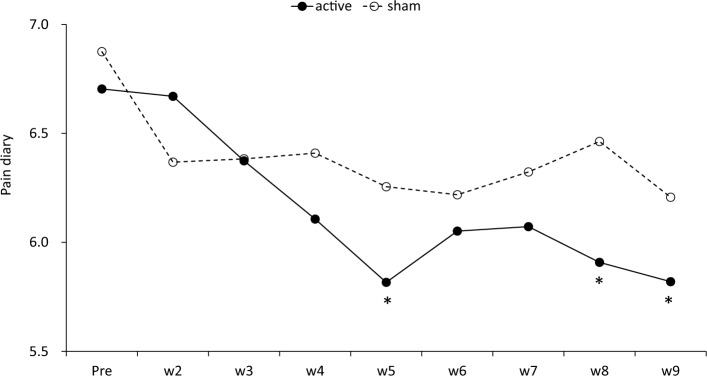
Time course of the average values of the pain diary in the intention-to-treat population. Asterisks indicate significant differences between pre (baseline), and each time point by paired *t*-test. W, week.

Figure 3 shows the time course of the average values in the pain diary of the intention-to-treat population. The decrease (least square [LS] means ± standard error [SE]) in the weekly average of the pain diary at week 9 compared to the baseline was 0.84 ± 0.31 in the active rTMS group and 0.58 ± 0.29 in the sham group (between-group mean difference, 0.26; 95% confidence interval [CI], − 0.60 to 1.13; *p* = 0.54). There was no significant effect on the interaction between the treatment group and the time point (*p* = 0.35) (Supplementary Table S2). The number of patients with ≥ 2 decrease in the pain diary was 2 (14%) in the active rTMS group and 2 (13%) in the sham, and the number of patients with ≥ 4 decrease was 2 (14%) and 0, respectively. The results of the subgroup analyses of the decrease in the pain diary are shown in Supplementary Fig. S1. Higher weekly average of pain diary and EQ-5D-5L scores, and lower SF-MPQ2 and PCS scores could be related to favorable outcomes.

### VASpre and SF-MPQ2

The decrease (LS means ± SE) in VAS_pre_ at week 9 compared to the baseline was 12.7 ± 3.5 in the active rTMS group and 9.7 ± 3.3 in the sham group (between-group mean difference, 2.9; 95% CI − 6.7 to 12.5; *p* = 0.54). There was no significant effect on the interaction between the treatment group and the time point (*p* = 0.29) (Supplementary Table S2). The number of patients with a ≥ 20 decrease in VAS_pre_ was 4 (29%) in the active rTMS group and 4 (25%) in the sham, and the number of patients with ≥ 40 decrease was 2 (14%) and 1 (6%), respectively. The decrease (LS means ± SE) in SF-MPQ2 at week 9 compared to the baseline was 9.8 ± 4.7 in the active rTMS group and 21.6 ± 4.4 in the sham group (between-group mean difference, − 11.8; 95% CI − 24.5 to 1.0; *p* = 0.07). There was no significant effect on the interaction between the treatment group and the time point (*p* = 0.44) (Supplementary Table S2).

### PGIC

**Figure 4:**
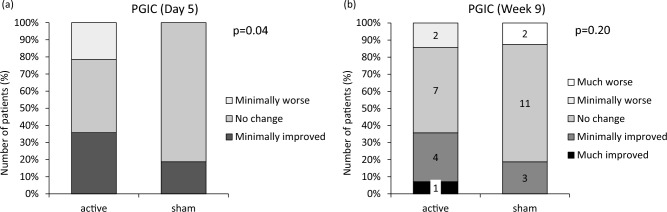
Distribution of patient improvement as measured by PGIC at (**a**) day 5 and (**b**) week 9. PGIC, Patient Global Impression of Change.

The number of patients (%) in the PGIC at day 5 was 5 (36%), 6 (43%), and 3 (21%) with “minimally improved,” “no change,” and “minimally worse” in the active rTMS and 3 (19%), 13 (81%), and 0 in sham stimulation (*p* = 0.04) (Fig. 4a). The number of patients (%) in PGIC at week 9 was 1 (7%), 4 (29%), 7 (50%), 2 (14%), and 0 with “much improved,” “minimally improved,” “no change,” “minimally worse,” and “much worse” in the active rTMS and 0, 3 (19%), 11 (69%), 0, and 2 (13%) in sham stimulation, respectively (*p* = 0.20) (Fig. 4b).

### Pain-related disability, cognitive function, QOL, and psychological conditions

PDAS decrease (LS means) was 0.8 (95% CI 2.8–13.3) in the active rTMS, and − 0.3 (− 5.2 to 4.6) in sham stimulation (*p* = 0.03). In contrast, changes in the PCS, EQ-5D-5L, BDI-II, and MMSE scores showed no significant differences between the groups (Table 2) (see details in Supplemental Table S3 for pre- and post-intervention values of these assessments).

**Table 2:** Comparison of the amount of change in clinical assessment such as psychology, ADL, and QOL before and after intervention based on ANCOVA.

	Active	Sham	*P*-value
PDAS	8.0 (2.8–13.3)	− 0.3 (− 5.2 to 4.6)	0.03
PCS			
Total	0.8 (− 2.2 to 3.8)	− 0.6 (− 3.4 to 2.1)	0.48
Rumination	1.1 (− 0.3 to 2.6)	− 0.3 (− 1.7 to 1.0)	0.15
Helplessness	− 0.2 (− 1.7 to 1.3)	0.5 (− 0.9 to 1.9)	0.50
Magnification	− 0.2 (− 1.0 to 0.7)	− 0.8 (− 1.6 to 0.0)	0.26
EQ-5D-5L	0.054 (− 0.02 to 0.13)	0.016 (− 0.05 to 0.08)	0.43
BDI-II	− 1.9 (− 5.7 to 1.9)	1.1 (− 2.4 to 4.7)	0.25
MMSE	0.6 (− 0.1 to 1.2)	− 0.02 (− 0.6 to 0.5)	0.17

Data are presented as the least-squares mean (95% confidence interval) of the decrease or increase in values at week 9 compared to baseline values. Before- and after-intervention comparisons for each assessment show PCS reduction, PDAS reduction, EQ-5D-5L increase, BDI-II reduction, and MMSE increase, meaning the degree of improvement.

ANCOVA, Analysis of covariance; PDAS, Pain Disability Assessment Scale; PCS, Pain Catastrophizing Scale; EQ-5D-5L, European Quality of Life-5 Dimensions 5-level; BDI-II, Beck Depression Inventory second version; MMSE, Mini-Mental State Examination.

### Response in VAS_post_ immediately after the intervention during the induction phase

The response to induction treatment in predicting efficacy at week 9 in the active rTMS group was investigated, and no association was found (Table S4).

### Adverse events and blinding

No serious adverse events were observed during the trial. However, four minor adverse events occurred, all of which were assessed by a blinded assessor as transient symptoms and were not directly related to the intervention. The adverse events were fever in the active rTMS group, and scalp pain, dizziness, and worsening of pain in the sham group, each occurring in one case (Table 3).

**Table 3:** Adverse events and blinding.

	Active (n = 14)	Sham (n = 16)
Adverse events	1 (7%) Fever	3 (19%) Scalp pain Dizziness Worsening of pain
Blinding		
Thought that they received active	5 (36%)	7 (44%)
Thought that they received sham	4 (29%)	4 (25%)
Cannot tell	5 (36%)	5 (31%)

Data are presented as number of patients (%).

In the blinded assessment, 36% of the patients in the active rTMS group and 44% in the sham stimulation group thought they received active stimulation; in contrast, 29% of the patients in the active rTMS group and 25% in the sham stimulation group thought they received active stimulation. 36% of patients in the active rTMS group and 31% in the sham stimulation group thought that they could not know the stimulation conditions (Table 3).

## Discussion

This randomized, patient- and assessor-blinded, sham-controlled, parallel trial evaluated the efficacy and safety of an eight-week administration of navigation-guided 10-Hz rTMS over M1, focusing on the target population of neuropathic pain in the upper limb. Although there was a tendency for better pain relief in the active rTMS group at week 9, the primary outcome, the decrease in a pain diary at each time point relative to the baseline, showed no interaction between the treatment group and time point. In other words, this study did not show significant pain relief for patients with neuropathic pain in the upper limbs compared to the sham, but active rTMS tended to provide better results than the sham stimulation. The PGIC tended to be better with active rTMS than with sham stimulation, and PDAS (pain-related disability) improved only in the active rTMS group. No serious adverse events were observed. Previous studies have recommended long-term administration and high-dose stimulation parameters of rTMS treatment for neuropathic pain, and this study confirmed these recommendations.

Based on the results of our previous study^20,23,26^, the stimulation parameters were modified from our previous study (5-Hz, 500 pulses per session, 5–10 daily sessions) in the present study (10-Hz, 2000 pulses per session, 12 sessions in total over 8 weeks). This is because we considered that higher frequency rTMS over the M1 with longer-term administration for neuropathic pain in the upper limbs would provide better pain relief. However, this study did not show positive results for primary outcomes. Compared with the results of previous studies, the pain-relieving effect of active rTMS in this study was inferior to those of the latest studies^33,49^, and there were notable differences in the assessments of pain intensity and the method of sham stimulation between many previous studies on rTMS for chronic pain and our group. First, the assessment of pain intensity differed between previous studies and our trials. Many previous studies have defined pain intensity as the change in value from baseline to the time point immediately after each intervention. In contrast, in this study, pain intensity was measured daily to accurately assess the pain status. This difference in the assessment of pain intensity may make a difference in the effect of rTMS and may need to be considered in the assessment method. We believe that our method of assessing daily pain intensity allows an accurate assessment of the pain intensity of a patient. Second, many RCTs on rTMS for chronic pain used sham coils and no stimulation sensation in the sham condition, whereas we used both sham coils and electrical stimulation to provide a stimulation sensation to the scalp similar to that of rTMS. Appropriate blinding was confirmed through a questionnaire evaluation. Only Attal et al. described how they performed low-intensity electrical stimulation on the head with surface electrodes via an electrical system; however, they did not describe the intensity or method of electrical stimulation^33^. Different sham conditions are likely to provide different placebo effects, and it is widely known that the placebo effect of interventions is pronounced in a variety of disorders, including pain, depression, and Parkinson’s disease^50–52^. Therefore, we must carefully consider the impact of the sham conditions. For example, the electrical stimulation used in this study can produce scalp sensations similar to active rTMS but is thought to produce very little or no stimulation to the brain due to the high electrical resistance of the skull. Therefore, similar electrical stimulation techniques have been used as sham stimulation in rTMS studies on Parkinson’s disease^53,54^, neuropathic pain^20,23,24^, and depression^55,56^. However, the pathogenesis of pain differs from those of Parkinson’s disease and depression. Nociceptive signals are transmitted to the central nervous system mainly through thin nerve fibers such as A-delta and C fibers; however, pain can be suppressed by inhibiting the signals of thin nerve fibers by stimulating thick nerves such as A-beta fibers (gate control theory)^57^. Additionally, it has recently been reported that migraines can be alleviated by a technique called remote electrical neuromodulation, which involves electrical stimulation of the upper limbs at a remote site that is different from the affected area^13,58,59^. Based on the results of our previous studies, we cannot rule out the possibility that electrical stimulation of the scalp may have caused the decreased pain. Furthermore, the open versus hidden administration group of morphine treatment for postoperative pain has been reported to produce pain relief in both conditions; however, the hidden group had less pain relief than the open group. This implies that knowledge about the treatment method can affect the outcomes if patients are not properly blinded to the sham condition. In other words, many previous studies may not have been properly blinded to the sham condition by using sham coils without stimulus sensation. Therefore, it may be necessary to discuss the method and assessment of sham stimulation in future studies of rTMS for neuropathic pain.

Since the results of this study are contrary to those of many previous studies, systematic reviews, and meta-analyses of rTMS treatment for neuropathic pain, it is necessary to consider why active rTMS in this study was insufficient with respect to its efficacy in pain relief. First, this study included fewer sessions and shorter intervention periods than recent studies^31–33^. Recently, a parallel-group RCT of rTMS for peripheral neuropathic pain was conducted with 15 sessions over 22 weeks, and pain relief was achieved in approximately 4 weeks^33^. Similar to previous studies, active rTMS was more effective for pain relief than sham stimulation after 4 weeks in the pain diary used in this study. Accordingly, further investigation of the effect of rTMS may be necessary with a longer intervention period and a greater number of sessions than the number of intervention periods and sessions used in this study (8 weeks). Second, it has been suggested that the efficacy of rTMS may differ between peripheral and central neuropathic pain. Reportedly, rTMS treatment is more effective for peripheral neuropathic pain than for central pain^19^. Furthermore, in a recent RCT, the efficacy of rTMS was demonstrated in selected patients with peripheral neuropathic pain^33^. We speculated that rTMS may be more effective in patients with peripheral neuropathic pain. However, one recent study that included only patients with central neuropathic pain reported the efficacy of rTMS^49^, and one review indicated that the efficacy of rTMS in patients with central neuropathic pain was superior to that in patients with peripheral neuropathic pain^17^. Currently, there is no consensus on whether rTMS treatment is more effective for central or peripheral neuropathic pain, and this issue requires further study. Third, the effects of rTMS may have been influenced by multiple sites of bilateral and unilateral pain. This is because rTMS is not effective at multiple sites or bilaterally for neuropathic pain after spinal cord injury^60^. Furthermore, the subgroup analysis in this study showed a trend toward slightly greater pain relief on the unilateral side than on the bilateral side. In addition, many previous studies have included patients with unilateral upper limb^49,61–67^. Therefore, unilateral neuropathic pain may be a more suitable target for rTMS treatment than bilateral pain.

This study found that PGIC scores and improvements in PDAS (pain-related disability) tended to be better with active rTMS than with sham stimulation. In recent years, while it has become important to reduce pain intensity in pain treatment, it has become more important to focus on improving patients’ QOL and activities of daily living (ADL). However, the best of our knowledge, a change in ADL, cognitive function, QOL, and psychological conditions before and after the intervention has rarely been reported in many previous studies of rTMS for neuropathic pain^23,33,60^. Although it is difficult to explain why rTMS treatment over M1 improves pain-related disability, it may affect not only pain intensity but also pain-related disability directly and indirectly related. We hope that the findings of this study will provide useful information for future studies indicating the possibility of improving the surrounding conditions in patients with neuropathic pain.

This study has several limitations. The number of patients, duration of intervention periods, and total number of sessions were small, and the study was conducted at a single center. Due to the small number of patients, there is naturally a concern that the statistical power of the study will be reduced. It is possible that the duration of the intervention and the small number of sessions made it difficult to determine the analgesic effect of rTMS. It is also difficult to generalize the results of this study because it was conducted at a single center. Finally, pain intensity was measured daily to accurately assess the effect of long-term administration of rTMS on neuropathic pain in the upper limb. To ensure the quality of this study, all data management, monitoring, and statistical analysis were performed by an independent organization. And this study was able to replicate, to some extent, the pain-relieving effect of long-term administration of rTMS for neuropathic pain suggested in previous studies. Therefore, the findings of this study can provide important basic data for designing future clinical trials.

## Conclusion

This study was a preliminary investigation of the efficacy and safety of rTMS in patients with neuropathic pain in the upper limb, considering various factors, including enrolled patients, stimulation parameters, and a number of interventions, based on the findings of previous studies. Pain diary and other pain scores did not show a significant pain relief effect for rTMS treatment in this study. However, PGIC tended to be better with active rTMS than with sham stimulation, and pain-related disability improved. Further studies are needed to confirm whether rTMS treatment is effective for the treatment of neuropathic pain.

### Supplementary Information

Supplementary Information.

## Data Availability

The datasets generated during the current study are available from the corresponding author on reasonable request.
