# Supplementary material for: Repetitive transcranial magnetic stimulation focusing on patients with neuropathic pain in the upper limb: a randomized sham-controlled parallel trial
Source: Sci Rep. 2024 May 23;14:11811. doi: 10.1038/s41598-024-62018-x (PMC11116497; doi:10.1038/s41598-024-62018-x)

| Supplementary Table S1. Patients’ current pain medication regimes | | |  |
| --- | --- | --- | --- |
|  | Total (30) | Active (n = 14) | Sham (n = 16) |
| Current pain medication available (%) | 29 (97%) | 14 (100%) | 15 (94%) |
| Current medication regime (%) |  |  |  |
| Pregabalin | 16 | 6 | 10 |
| Gabapentin | 2 | 1 | 1 |
| Mirogabalin | 3 | 3 | 0 |
| SNRI (Duloxetine) | 9 | 6 | 3 |
| TCA (Amitriptyline) | 6 | 2 | 4 |
| TCA (Nortriptyline) | 1 | 1 | 0 |
| Tramadol | 11 | 4 | 7 |
| Neurotropin | 5 | 4 | 1 |
| Baclofen | 2 | 1 | 1 |
| Clonazepam | 5 | 2 | 3 |
| Acetaminophen | 10 | 3 | 7 |
| Loxoprofen | 1 | 0 | 1 |
| Mecobalamin | 2 | 2 | 0 |
| Buprenorphine | 1 | 1 | 0 |
| Buprenorphine Hydrochloride | 1 | 0 | 1 |
| Bromazepam | 1 | 1 | 0 |
| Others | 1 | 1 | 0 |
| SNRI, Serotonin Noradrenaline Reuptake Inhibitors; TCA, Tricyclic antidepressants. | | | |

| Supplementary Table S2. Time courses of treatment effect of rTMS on each pain intensity scores (raw data) | | | | | | | | | | | | |
| --- | --- | --- | --- | --- | --- | --- | --- | --- | --- | --- | --- | --- |
| ***Pain diary*** | |  |  |  |  |  |  |  |  |  |  |  |
|  | Baseline | 2^nd^ week | 3^rd^ week | 4^th^ week | 5^th^ week | 6^th^ week | 7^th^ week | 8^th^ week | 9^th^ week |  |  |  |
| Active | 14 | 14 | 14 | 13 | 13 | 13 | 13 | 13 | 13 |  |  |  |
|  | 6.7 (1.0) | 6.7 (1.1) | 6.4 (1.3) | 6.1 (1.5) | 5.8 (1.9) | 6.1 (1.8) | 6.1 (1.8) | 5.9 (1.8) | 5.8 (2.0) |  |  |  |
| Sham | 16 | 16 | 16 | 15 | 15 | 15 | 15 | 15 | 15 |  |  |  |
|  | 6.9 (1.3) | 6.4 (1.5) | 6.4 (1.7) | 6.4 (1.7) | 6.3 (1.6) | 6.2 (1.8) | 6.3 (1.6) | 6.5 (1.7) | 6.2 (1.8) |  |  |  |
| ***VAS*** |  |  |  |  |  |  |  |  |  |  |  |  |
|  | Baseline | 2^nd^ week | 3^rd^ week | 4^th^ week | 5^th^ week | 6^th^ week | 7^th^ week | 8^th^ week | 9^th^ week | 10^th^ week | 12^th^ week | 16^th^ week |
| Active | 14 | 14 | 14 | 13 | 13 | 12 | 13 | 13 | 13 | 12 | 13 | 13 |
|  | 67.6 (12.1) | 65.2 (14.5) | 68.6 (16.6) | 60.8 (15.8) | 58.8 (18.2) | 61.6 (17.0) | 59.2 (20.3) | 58.2 (18.3) | 54.3 (21.1) | 56.2 (22.7) | 58.0 (22.4) | 53.8 (24.5) |
| Sham | 16 | 16 | 16 | 15 | 15 | 15 | 15 | 15 | 15 | 15 | 14 | 15 |
|  | 68.9 (14.0) | 59.9 (19.5) | 61.1 (18.6) | 61.1 (18.8) | 59.3 (21.4) | 60.2 (19.2) | 58.1 (19.6) | 61.9 (19.5) | 58.6 (22.2) | 62.1 (21.5) | 60.9 (22.4) | 64.3 (18.4) |
| ***SF-MPQ2*** | |  |  |  |  |  |  |  |  |  |  |  |
|  | Baseline | 2^nd^ week | 3^rd^ week | 4^th^ week | 5^th^ week | 6^th^ week | 7^th^ week | 8^th^ week | 9^th^ week | 10^th^ week | 12^th^ week | 16^th^ week |
| Active | 14 | 14 | 14 | 13 | 13 | 12 | 13 | 13 | 13 | 12 | 13 | 13 |
|  | 72.4 (50.9) | 66.1 (54.6) | 72.0 (58.7) | 57.6 (55.2) | 58.9 (57.0) | 63.2 (59.0) | 58.2 (59.4) | 60.5 (59.4) | 57.8 (58.0) | 57.3 (61.8) | 59.7 (59.2) | 57.3 (60.8) |
| Sham | 16 | 16 | 16 | 15 | 15 | 15 | 15 | 15 | 15 | 15 | 14 | 15 |
|  | 87.5 (46.2) | 75.5 (43.0) | 74.3 (42.8) | 77.2 (44.1) | 68.9 (43.7) | 72.3 (48.5) | 73.1 (47.3) | 73.4 (45.9) | 68.4 (47.2) | 74.0 (47.5) | 81.2 (54.8) | 79.8 (48.2) |

Data are presented as number of patients and mean (standard deviation).

| Supplementary Table S3. Outcomes of clinical assessments, including psychological, ADL, and quality of life | | | | |
| --- | --- | --- | --- | --- |
|  | Active | | Sham | |
|  | Pre-intervention | Post-intervention | Pre-intervention | Post-intervention |
| PDAS | 24.8 (14.2) | 15.8 (10.0) | 22.0 (13.9) | 23.1 (11.0) |
| PCS |  |  |  |  |
| Total | 30.6 (9.4) | 29.9 (12.6) | 30.8 (9.2) | 31.4 (6.3) |
| Rumination | 14.6 (3.8) | 13.6 (5.4) | 15.6 (3.8) | 15.8 (2.7) |
| Helplessness | 10.5 (4.7) | 10.7 (5.6) | 10.6 (3.4) | 10.1 (3.2) |
| Magnification | 5.5 (3.1) | 5.6 (3.6) | 4.7 (2.8) | 5.6 (2.0) |
| EQ-5D-5L | 0.586 (0.16) | 0.636 (0.20) | 0.521 (0.20) | 0.541 (0.21) |
| BDI-II | 12.9 (9.9) | 15.1 (12.5) | 16.4 (7.7) | 14.9 (6.6) |
| MMSE | 28.9 (1.7) | 29.5 (1.4) | 29.4 (1.3) | 29.3 (1.4) |
| Data shows mean (standard deviation)  PDAS, Pain Disability Assessment Scale; PCS, Pain Catastrophizing Scale; EQ-5D-5L, European Quality of Life-5 Dimensions 5-level; BDI-II, Beck Depression Inventory second version; MMSE, Mini-Mental State Examination. | | | | |

| Supplementary Table S4. Correlation between response during the induction period and efficacy at week 9 of the pain diary in the active rTMS group | | |
| --- | --- | --- |
|  | Correlation coefficient | p-value |
| Day 1 | 0.37 | 0.20 |
| Day 1-2 | 0.19 | 0.50 |
| Day 1-3 | 0.12 | 0.70 |
| Day 1-4 | 0.08 | 0.78 |
| Day 1-5 | 0.17 | 0.57 |

Supplementary Figure 1. Details of the schedule for the evaluation of the study

| Time | W-4 | D-6 |  | D1 | D2 | D3 | D4 | D5 | W2 | W3 | W4 | W5 | W6 | W7 | W8 | W9 | W10 | W12 | W16 |
| --- | --- | --- | --- | --- | --- | --- | --- | --- | --- | --- | --- | --- | --- | --- | --- | --- | --- | --- | --- |
| Pain diary |  |  |  |  |  |  |  |  |  |  |  |  |  |  |  |  |  |  |  |
| VAS, SF-MPQ2^*^ | X |  |  |  | X | X | X | X | X | X | X | X | X | X | X | X | X | X | X |
| PGIC |  |  |  |  |  |  |  | X |  |  |  |  |  |  |  | X |  |  |  |
| PCS, PDAS,  EQ-5D-5L |  |  |  | X |  |  |  |  |  |  |  |  |  |  |  | X |  |  |  |
| BDI-II, MMSE | X |  |  |  |  |  |  |  |  |  |  |  |  |  |  | X |  |  |  |

A Pain diary was kept every morning at home to evaluate average pain intensity over the past 24 hours from the pre-intervention period to one week after the end of the intervention (week 9).

* VAS and SF-MPQ2 scores were also assessed for average pain over the past 24 h immediately before each intervention during the intervention period at weeks 9, 10, 12, and 16. Immediately after each intervention, the VAS score was used to assess the current pain.

BDI, Beck Depression Inventory-Second Edition; D, day; EQ-5D, EuroQol-5 Dimension; MMSE, Mini Mental State Examination; PCS, Pain Catastrophizing Scale; PDAS, Pain Disability Assessment Scale; PGIC, Patient Global Impression of Change; rTMS, repetitive transcranial magnetic stimulation; W, week.

Supplementary Figure 2


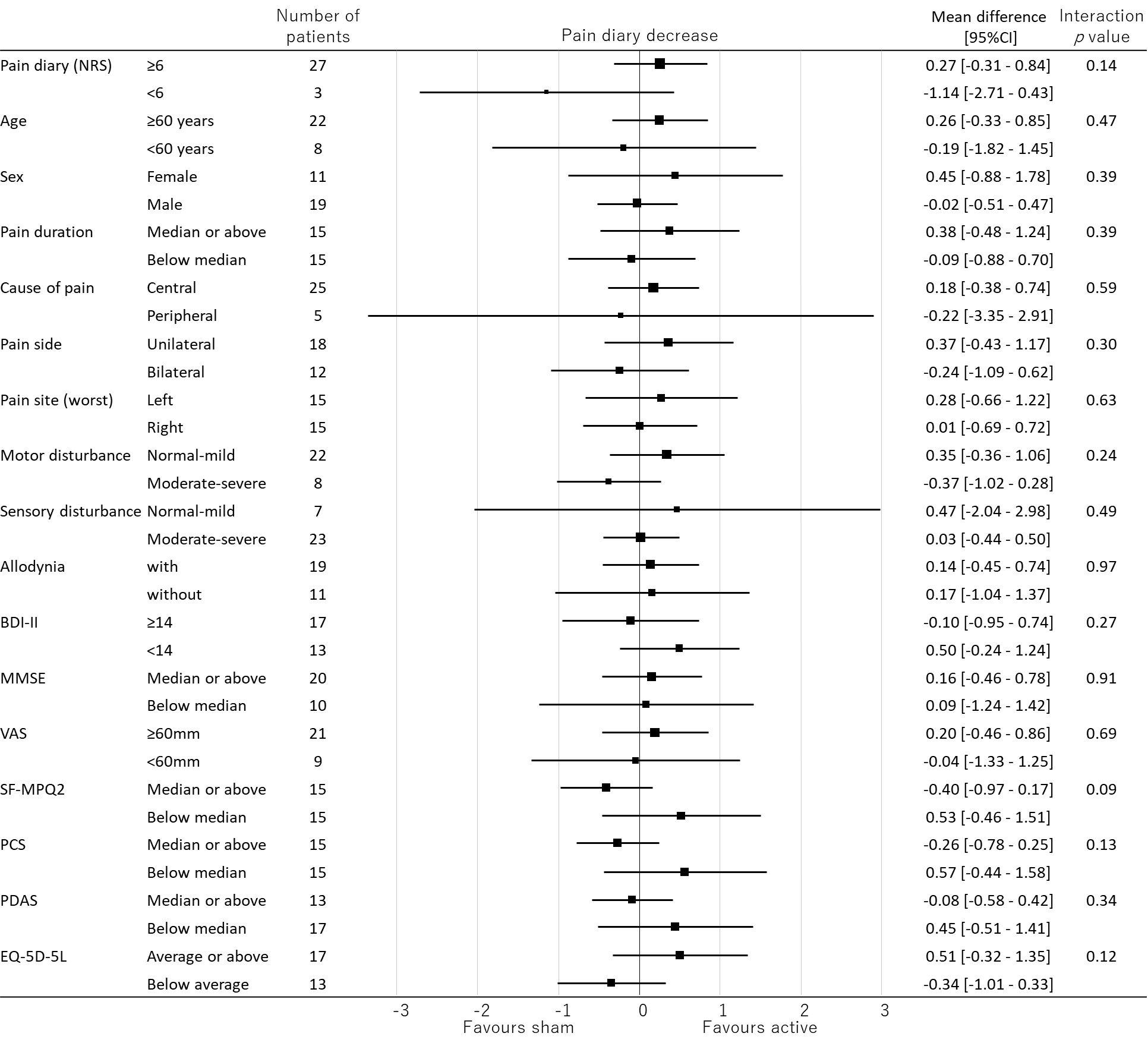

Supplement: Supplementary file 1 — Supplementary Information. [file 41598_2024_62018_MOESM1_ESM.docx]
